# The immune adherence receptor CR1-like existed on porcine erythrocytes membrane

**DOI:** 10.1038/srep13290

**Published:** 2015-08-13

**Authors:** Wei Yin, Jiaoyan Cui, Junbing Jiang, Junxing Zhao, Kuohai Fan, Na Sun, Zhiwei Wang, Yaogui Sun, Haili Ma, Hongquan Li

**Affiliations:** 1 College of Animal Science and Veterinary Medicine, Shanxi Agriculture University, Taigu, Shanxi 030801, P. R. China

## Abstract

In the present study, we obtain a mouse anti-porcine complement receptor type 1 (CR1)-like monoclonal antibody (McAb) and use this McAb to verify the existence of CR1-like protein on porcine erythrocytes. Our results confirm that CR1-like protein is localized on the surface of porcine erythrocytes. Mouse immunoglobulin G inhibited the binding of serum-opsonized green fluorescent protein-expressing *Escherichia coli* to porcine erythrocytes. Sodium dodecyl sulfate-polyacrylamide gel electrophoresis analysis indicates that CR1-like McAb reacts with biochemically-purified porcine erythrocyte membrane fractions, with a clear band at 135 kDa to 140 kDa. We postulate that the 135 kDa to 140 kDa membrane protein is the equivalent of the porcine erythrocyte CR1-like protein.

Human erythrocyte complement receptor type 1 (CR1), also known as CD35, is a large single-chain transmembrane glycoprotein and an immune adherence (IA) receptor of complement component 3b/4b (C3b/C4b)^1^,^2^. In primates, erythrocytes bind C3b, C4b, or C1q-bearing immune complexes (ICs) to their membranes via CR1^3^. Furthermore, binding of CR1 to complement components induces phagocytosis of opsonized ICs^4^,^5^. Therefore, the IA of primate erythrocytes plays an important role in the clearance of ICs from the circulation. However, the activity of erythrocyte IA (E-IA) in nonprimates is still controversial, and if it does occur, whether it is mediated by activated complements on ICs interacting with CR1 on erythrocytes remains unclear.

Our previous studies demonstrated that the activity of E-IA in chickens with infectious bursal disease virus was significantly reduced^6^ and *Astragalus* polysaccharides increased E-IA in mice with adjuvant-induced arthritis and reduced the deposition of ICs in joint synovium^7^. Using fluorescence microscopy, scanning electron microscopy (SEM), and transmission electron microscopy (TEM), we also demonstrated that E-IA occurs in porcine erythrocytes^8^. In addition, a full-length porcine CR1-like cDNA has been cloned and analyzed bioinformatically, and the sequence has been submitted to GeneBank (Accession number: AGU01766.1)^9^. The objective of the current study was to prepare anti-porcine CR1-like monoclonal antibody (McAb) to further verify the existence of porcine CR1-like protein on erythrocyte membranes.

## Results

### Indirect immunofluorescence observation of CR1-like protein on porcine erythrocytes

**Figure 1:**
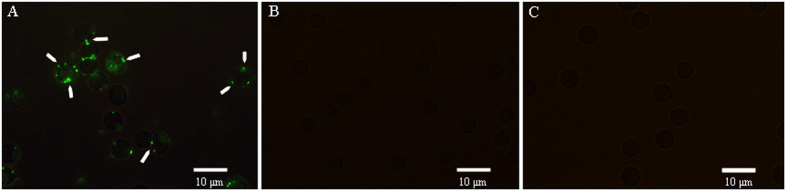
Representative examples of the interaction between CR1-like McAb and porcine erythrocyte CR1-like protein. Green fluorescence was observed on the surface of erythrocytes (**a**) but no fluorescence was observed in the absence of CR1-like McAb (**b** and **c**). Oil microscopy magnification is 1000 ×.

CR1-like McAb and goat anti-mouse IgG-FITC are used to perform the indirect immunofluorescence assay for porcine erythrocyte CR1-like protein. Green fluorescence is clearly seen on the surface of the porcine erythrocytes (Fig. 1A). When mouse IgG is substituted for CR1-like McAb as the first antibody, no fluorescence is observed (Fig. 1B). Furthermore, no fluorescence is observed when the erythrocytes are treated with goat anti-mouse IgG-FITC alone (Fig. 1C). These results clearly indicate the existence of CR1-like protein on the surface of porcine erythrocytes.

### Blocking assay of porcine erythrocyte immune adherence

**Figure 2:**
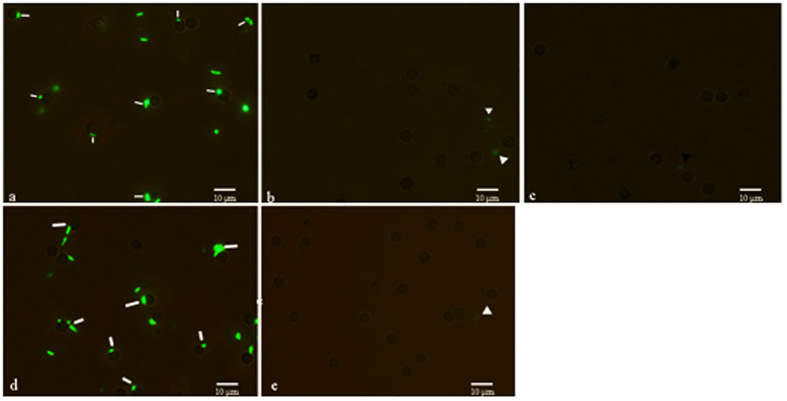
Representative examples of the interaction between porcine erythrocytes and GFP-*E. coli* under different experimental conditions. The adherence of opsonized GFP*-E. coli* to erythrocytes is indicated by the white bars (**a** and **d**). The white and black triangles indicate that there was no adherence of opsonized GFP-*E. coli* (**b,c** and **e**). Oil microscopy magnification is 1000 ×.

Porcine erythrocytes are incubated with green fluorescent protein-expressing (GFP)-*Escherichia coli* opsonized in normal serum, non-opsonized GFP-*E. coli*, and GFP-*E. coli* opsonized in ethylenediaminetetraacetic acid (EDTA)-treated serum. Opsonized GFP-*E. coli* adheres to the porcine erythrocytes (Fig. 2a), and non-opsonized GFP-*E. coli* is seen on the surface of the erythrocytes (Fig. 2b). However, there was no fluorescence observed on the erythrocytes incubated with GFP-*E. coli* opsonized in EDTA-serum (Fig. 2c). These data suggest the necessity of normal serum in the process of IA.

To test whether CR1-like protein is necessary for the binding of opsonized GFP-*E. coli* on porcine erythrocytes, CR1-like McAb and mouse IgG are added into the incubation system. As shown in Fig. 2d,e, no fluorescence is observed on the surface of the porcine erythrocytes when CR1-like McAb is added, but fluorescence appears when mouse IgG is added. These results demonstrate that opsonized GFP-*E. coli* adheres to porcine erythrocytes via the CR1-like protein located on the erythrocyte membrane.

### Statistical analysis of porcine E-IA Blocking Assay Data

The experimental blood samples are divided into five groups, a to e, as shown in Table 1. There are more positive erythrocytes in group a compared with groups b and c (Table 2), indicating that IA ability differs among these three groups. There is a positive correlation between erythrocyte IA and serum levels of C3 (*P* < 0.05). There are fewer positive erythrocytes in group d compared with groups a and e (Table 3), and erythrocyte IA is positively correlated with levels of CR1-like protein on the erythrocyte surface (*P* *<* 0.05).

### Immunoprecipitation assay of porcine erythrocyte CR1-like protein

**Figure 3:**
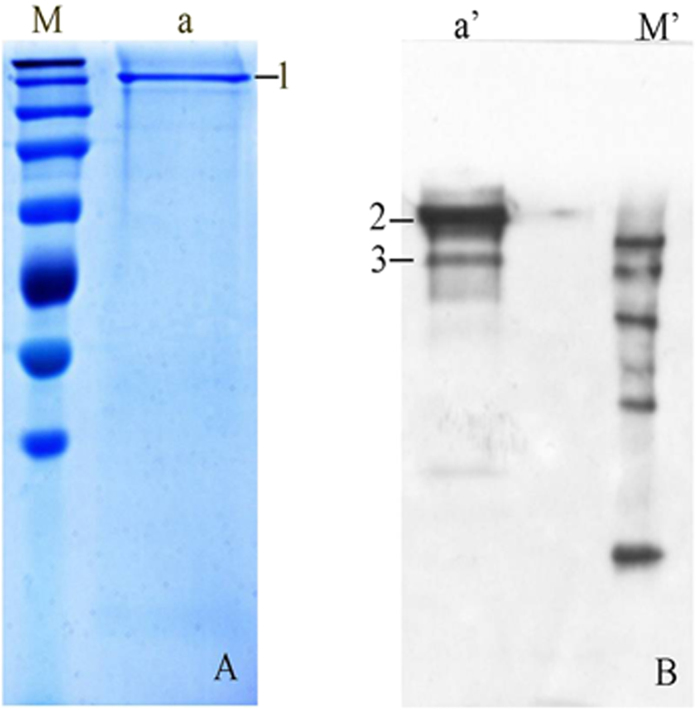
SDS-PAGE and Western blot analysis of porcine erythrocyte CR1-like protein under nonreduced conditions. Line **a** shows the porcine erythrocyte CR1-like protein band on SDS-PAGE, and lines labeled **M** are protein markers (from top): 300 kDa, 250 kDa, 180 kDa, 130 kDa, 100 kDa, 70 kDa, and 50 kDa (**a**). Line **a** shows the porcine erythrocyte CR1-like protein band on Western blot, and lines labeled **M** are protein markers (from top): 150 kDa, 120 kDa, 90 kDa, 60 kDa, and 40 kDa (**b**).

The CR1-like protein is isolated from porcine erythrocytes by immunoprecipitation (IP) assay and the eluted sample is analyzed using sodium dodecyl sulfate-polyacrylamide gel electrophoresis (SDS-PAGE) (Fig. 3a) and Western blot (Fig. 3b). Under nonreduced conditions, one major band is seen at approximately 250 kDa (1^#^ band) on SDS-PAGE, and this band demonstrates the specificity of the CR1-like McAb. On Western blot analysis, one major band at 200 kDa and a minor band at 140 kDa (2^#^ and 3^#^ bands) are seen.

**Figure 4:**
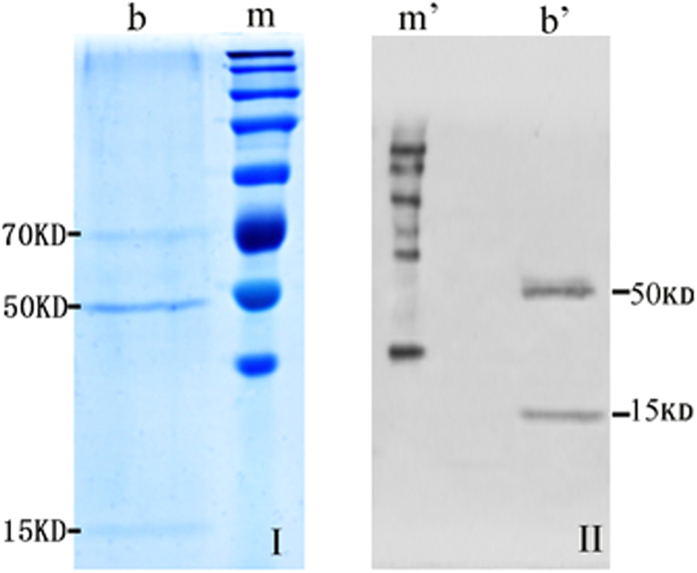
SDS-PAGE electrophoresis and Western blot for porcine erythrocyte CR1-like protein under reduced conditions. Line **b** shows the porcine erythrocyte CR1-like protein band on reduced-SDS-PAGE, and line **m** is a protein marker (**a**). Line **b** shows the CR1-like protein band on Western blot, and line **m** is a protein marker.

The results under nonreduced conditions indicate that the molecular weight of the CR1-like protein on porcine erythrocytes is in the range of 140 kDa to 200 kDa. Under reduced conditions, three bands are seen (at 70 kDa, 50 kDa, and15 kDa; Fig. 4a), of which two (50 kDa, 15 kDa) are further confirmed by Western blot analysis (Fig. 4b). This suggests that the CR1-like protein on porcine erythrocytes contains at least one disulfide bond. The antigen binding sites are located in the 50 kDa and 15 kDa peptide fragments. Therefore, it is likely that porcine erythrocyte CR1-like protein contains more than one subunit.

## Discussion

In humans and primates, CR1 is a multifunctional polymorphic glycoprotein that exists on the membranes of various cells, including erythrocytes, eosinophils, monocytes, macrophages, B-lymphocytes, dendritic cells, Langerhan cells, and glomerular podocytes. The *CR1* gene has been studied in many different species because of its important role in mammalian immune system activation. It is now clear that CR1 can bind C3b, a cleavage product of C3 in the serum, through multiple binding domains^10^. Erythrocytes in nonhuman primates have been shown to have a functional CR1-like protein, which has the ability to bind to C3b. For example, baboon erythrocytes have a 65 kDa C3b-binding protein with functional characteristics similar to human CR1^11^. Furthermore, a previous study identified a 75 kDa protein in chimpanzee erythrocytes, which has an IA function^12^. In nonprimate animals, the existence of an IA function of erythrocytes remains controversial. Till date, a CR1-like protein has not been clearly identified in nonprimate animals.

In our previous study, using SEM and TEM we demonstrated IA of C3b-oposinized GFP-*E. coli* in porcine erythrocytes^8^, indicating that erythrocytes have an IA function in nonprimate animals, at least in porcine species. Complement regulatory proteins, like CR1, are known to play an important role in human and primate erythrocyte IA. However, this function has not been clearly demonstrated in other species. In particular, it remains to be determined whether similar complement regulatory proteins mediate the IA function in nonprimates. We have successfully cloned full sequences of CR1-like cDNA from porcine erythrocytes^9^, and predicted CR1-like protein structure and functional sites using bioinformatics. This played an important role in the preparation of the CR1-like McAb. In the current study, we first acquired porcine CR1-like McAb, produced based on its biologic information, from Beijing Protein Innovation Co., Ltd. (Beijing, China). The produced McAb met high quality standards^13^,^14^, including high purity and affinity. Further research on the immune function of porcine erythrocytes was conducted with the CR1-like McAb. Immunofluorescence assay clearly demonstrated antigenic cross-reactivity between CR1-like McAb and porcine erythrocytes, which further confirmed that a CR1-like protein exists on the surface of porcine erythrocytes (Fig. 1A–C). Furthermore, blocked assay (Fig. 2a,d and e) revealed that CR1-like McAb blocked binding of opsonized GFP-*E. coli* to the porcine erythrocytes, suggesting similar functions of the CR1-like protein and the C3b receptor.

In the normal serum group (Fig. 2a), porcine erythrocytes showed IA to the GFP-*E. coli*, but IA was not observed in the group without serum (Fig. 2b), or in the EDTA-treated serum group (Fig. 2c). Therefore, porcine erythrocytes did not exhibit IA when serum was absent or inactivated. These results suggest that the IA of porcine erythrocytes via CR1-like protein is mediated by complement in the serum.

In addition, using IP and Western blot we demonstrated that the molecular weight of CR1-like protein in porcine erythrocytes was around 135 kDa to 140 kDa (Figs 3 and 4). This molecular weight differs from that reported for primate animals, including baboons and chimpanzees. Accordingly, we inferred that CR1 and CR1-like protein show structural variance among different species.

In conclusion, our data demonstrate the existence of CR1-like protein on the surface of porcine erythrocytes. This protein is the IA receptor for C3b and has a molecular weight of 135 kDa to 140 kDa. In terms of animal red blood cell immunity theory, there are many questions still to be answered. For example, are there *CR1-like* gene polymorphisms in nonprimate animals as there are *CR1* gene polymorphisms in humans and primates? Are there regular factors involved in the IA of porcine erythrocytes *in vivo*? What is the role of CR1-like protein in the immune system? The research in our laboratory is focused on answering these questions and we hope to be able to share the results in the near future.

## Methods

### Ethics Statement

All animals used in the present experiments were cared for humanely and the use of the animals was approved by the ethics committee at the Animal Science and Veterinary Medicine College of Shanxi Agriculture University in China. All experiments were conducted in compliance with the International Guiding Principles for Biomedical Research Involving Animals (CIOMS and ICLAS, December 2012).

### Animals

Five clinically healthy landrace pigs (22 ± 2.67 kg) were obtained from a commercial pig farm and housed under hygienic conditions at room temperature with good ventilation and free access to food and water. Two healthy adult rabbits were obtained from Shanxi Agricultural University. BALB/c female mice were provided by Beijing Protein Innovation Co., Ltd.

### Bacteria and Serum

GFP-*E. coli*, a genetically engineered bacteria strain expressing GFP, was produced in our laboratory^8^. Whole blood was drawn from the heart of anesthetized rabbits and serum was prepared by centrifuging at 206 × *g* for 10 min after standing at 37 °C for 5 min. Serum was freshly prepared for each batch of porcine erythrocytes.

### Preparation of Erythrocytes

Pigs were acclimatized to the new environment for at least one week before use. Whole blood (2 mL) was drawn from the precaval vein and placed in acid citrate dextrose anticoagulant tubes, and 0.5 mL of anticoagulated blood was diluted with an equal volume of PBS (pH 7.4). Erythrocytes were separated with lymphocyte separation medium (TBD Sciences, Tianjin, China) according to the manufacturer’s manual. Purity of the cells was confirmed by cell counting with a hemocytometer. The purified cells suspension was centrifuged and washed twice with isotonic (2.37%) sodium iodide (Sigma, St. Louis, MO, USA) to elute adsorbed serum proteins, and then resuspended in PBS for a concentration of 1 × 10^7^ cells mL^−1^.

### Preparation of Membrane Protein

The porcine erythrocyte membrane protein was obtained using a ReadyPrep™ Protein Extraction Kit (Membrane II) (Bio-Rad, USA) according to the manufacturer’s instructions. In brief, 50 μL phenylmethylsulfonyl fluoride (PMSF) (Kangwei Corp., Jinzhong, China), a 100 to 200 mg erythrocyte pellet, and 1 mL lysis buffer were mixed and treated using the supersonic schizolysis method. The lysate was centrifuged for 10 min at 2291 × *g* at 4 °C, and the supernatant was collected. Next, 60 mL enrichment reagent and 50 μL PMSF were added to the supernatant and stirred gently for 1 h. The precipitate was washed twice in lysis buffer by centrifuging for 1 h at 10,0000 × *g* at 4 °C. The membrane protein was dissolved in 1% SDS for SDS-PAGE.

### Preparation of Mouse Anti-Porcine CR1-like McAb

Complement control protein (CCP) sequences of porcine *CR1-like* (GeneBank ID: AGU01766.1) were analyzed using the conserved domain search tool (CD Search), and 19 CCPs were identified. These CCPs were synthetized by Beijing Protein Innovation Co., Ltd. and used to screen antigenic epitopes and prepare McAbs. Hybridoma cell lines secreting high titers of mouse anti-porcine CR1-like McAb (CR1-like McAb, in short) were injected into mice abdomen, and the peritoneal fluid was collected and purified using protein G affinity chromatography^9^,^10^ The purity, specify, and affinity of CR1-like McAb were verified by Beijing Protein Innovation Co., Ltd. using SDS-PAGE, Western blot, and ELISA, respectively. The purity was ≥95% and the final concentration was 3.12 mg/mL. SDS-PAGE showed that the molecular weight of the CR1-like McAb was around 150 kDa and the molecular weights of the heavy (49 KD) and light (26 KD) chains were also determined. ELISA results indicated that the affinity constant of the CR1-like McAb was 1.4E + 10.

### Complement-Opsonized Particles

The GFP-*E. coli* solution was divided into three parts. The first part was opsonized as described previously^8^. In brief, 1 mL of GFP-*E. coli* culture was incubated with 1 mL of fresh rabbit serum at 37 °C for 15 min in the dark in a shaking incubator with a rotational radius of 10 cm and then harvested by centrifuging at 2291 × *g* for 5 min. The bacterial pellet was washed twice with PBS by centrifuging at 2291 × *g* for 5 min and then resuspended in 1 mL of PBS (0.1 mol L^−1^, pH 7.4). The second part was used as a blank control, and did not receive any treatment. The third part was used as a negative control and was treated as follows: 30 μL of fresh rabbit serum and 20 μL of 2.5 mM EDTA were incubated at 37 °C for 10 min; then, 60 μL of GFP-*E. coli* culture was mixed with 40 μL of the EDTA-treated serum.

### Indirect Immunofluorescence Assay

The original solutions of CR1-like McAb and goat anti-mouse IgG-FITC (Beijing Com Win Biotech Co., Ltd.) were diluted at 1:50 before use. First, 20 μL of the original CR1-like McAb solution was added into 30 μL of erythrocyte suspension (1.5 × 10^7^ cells mL^−1^) and incubated for 30 min at 37 °C. The mixture was gently washed twice to remove unconjugated CR1-like McAb by centrifuging at 206 × *g* for 5 min and resuspended with 0.5 mL PBS. Subsequently, 15 μL goat anti-mouse IgG-FITC was added into the resuspension and the above procedures were repeated. Two control groups, groups I and II, were set up simultaneously for this assay. In group I, the erythrocyte suspension was treated with mouse IgG (Abcam, Cambridge, UK), and the above procedures were repeated. In group II, only 15 μL goat anti-mouse IgG-FITC was added into the erythrocyte suspension and incubated in dark. All erythrocyte samples were examined using a fluorescence microscope (BX51; Olympus, Tokyo, Japan).

### Blocking Assay of E-IA

As shown in Table 1, the experimental blood samples were divided into five groups, marked a, b, c, d and e. In group a, 50 μL porcine erythrocyte suspension was incubated with 50 μL opsonized GFP-*E. coli* solution at 37 °C for 1 h in a shaking incubator with a rotational radius of 10 cm. The mixture was washed twice in PBS by centrifuging at 206 × *g* for 5 min. In group b, the above steps were repeated with original GFP-*E. coli* instead of opsonized GFP-*E. coli*. In group c, 50 μL porcine erythrocyte suspension was treated with the GFP-*E. coli* opsonized by EDTA-treated serum; other steps were performed as for group a. In group d, the porcine erythrocyte suspension was incubated with 20 μL original CR1-like McAb solution at 37 °C for 1 h in a shaking incubator with a rotational radius of 10 cm and then washed twice in PBS by centrifuging at 206 × *g* for 5 min and resuspended in 0.5 mL PBS. Subsequently, 15 μL goat anti-mouse IgG-FITC was added into the resuspension. Next, 50 μL opsonized GFP-*E. coli* was added into the resuspended erythrocyte suspension, and washing steps were performed as for group a. In group e, all steps were same as in group d, except mouse IgG was used instead of CR1-like McAb. All samples were observed under a fluorescence microscope.

### Immunoprecipitation and SDS-PAGE

Latex beads (200 nm; 100 μL) were suspended in 1 mL coupling buffer (Kangwei Corp.) and mixed with 10 μL of CR1-like McAb; the pH was immediately adjusted to 6.5. Next, 100 μL of activation buffer (Kangwei Corp.) was added to the solution and the mixture was incubated for 15 min at room temperature in dark. The latex beads were then washed twice in coupling buffer and collected by centrifuging at 9761 × *g* for 5 min, and the bead pellet was resuspended with 500 μL coupling buffer. Finally, 1 mL membrane protein solution of porcine erythrocytes was coincubated with the latex beads at 4 °C overnight and then washed twice in coupling buffer and centrifuged at 9761 × *g* for 5 min.

The target protein eluted with spent regenerant (1 M NaCl, 0.1 M glycine, 8 M urea, 2% SDS) was subjected to nonreducing SDS-PAGE and reducing SDS-PAGE (5% spacer gel, 10% separation gel) to determine the target protein molecular weights. The power supply was set to 80 V for 0.5 h and 100 V for 3 h during electrophoresis. The separation gels were removed from the SDS-PAGE equipment and transferred onto PVDF membranes (0.22 μm; Merck Millipore, Germany). The transferred voltage was set to 60 V for 2.5 h at 4 °C. Next, the membrane was blocked with the blocking buffer containing 5% BSA (IgG-Free, Protease-Free; Jackson ImmunoResearch, Pennsylvania, USA) for 2 h at room temperature and washed twice with the TBS buffer for 5 min per time. The nonreducing and reducing membrane samples were then incubated with CR1-like McAb (1:1000) at 4 °C overnight. Goat anti-mouse IgG-HRP (1:10,000) was incubated with the membranes for 1 h at 37 °C and the protein bands were visualized using TMB substrate chromogenic agent (Elisatop Biotech, Beijing, China) and exposed to X-ray film.

### Statistical analysis of E-IA Blocking Assay Data

Erythrocytes were classified as positive if at least two green fluorescent spots were observed on the surface under fluorescence microscopy. Data were analyzed using the χ^2^ test of independence implemented in Microsoft Excel 2010 (Microsoft Corporation, USA). *P* < 0.05 was considered significant.

## Additional Information

**How to cite this article**: Yin, W. *et al.* The immune adherence receptor CR1-like existed on porcine erythrocytes membrane. *Sci. Rep.* **5**, 13290; doi: 10.1038/srep13290 (2015).

## Figures and Tables

**Table 1:** Experimental groups.

**Materials Groups**	**Erythrocytes**	**1-GFP-*****E. coli***	**2-GFP-*****E. coli***	**3-GFP-*****E. coli***	**CR1-like McAb**	**Mouse IgG**	**Second antibody**
a	+	+	/	/	/	/	/
b	+	/	+	/	/	/	/
c	+	/	/	+	/	/	/
d	+	+	/	/	+	/	+
e	+	+	/	/	/	+	+

**Notes** 1**-**GFP-*E. coli*: GFP-*E. coli* opsonized in normal serum; 2-GFP-*E. coli*: original GFP-*E. coli*; 3*-*GFP-*E. coli*: GFP-*E. coli* opsonized in EDTA-treated serum; Second antibody: goat anti-mouse IgG-FITC.

**Table 2:** Comparison among groups a, b, and c.

**Results Groups**	**Positive erythrocytes**	**Negative erythrocytes**	**Total erythrocytes**	**Theoretical positive number**	**Theoretical negative number**	**Total**
a	410	1997	2407	220.1438	2186.85617	2407
b	17	1180	1197	109.4774	1087.52257	1197
c	41	1472	1513	138.3787	1374.62126	1513
Total	468	4649	5117	468	4649	5117
Proportion	0.09146	0.90854	1	P = 6.56517E-75		

Data show the difference in adhesion ability under the various serum conditions. The adhesion ability in group a is stronger compared with groups b and c.

**Table 3:** Comparison among groups a, d, and e.

**Results Groups**	**Positive erythrocytes**	**Negative erythrocytes**	**Total erythrocytes**	**Theoretical positive number**	**Theoretical negative number**	**Total**
a	410	1997	2407	324.9524	2082.04765	2407
d	224	1935	2159	291.4716	1867.52841	2159
e	250	1732	1982	267.5761	1714.42395	1982
Total	884	5664	6548	884	5664	6548
Proportion	0.135003	0.864997	1	P = 1.59012E-10		

Data show the difference in adhesion ability under different antibody conditions. The adhesion ability in group d is lower compared with groups a and e.
